# Use of Castor Oil in Dermatology: A Narrative Review

**DOI:** 10.7759/cureus.103289

**Published:** 2026-02-09

**Authors:** Kathleen Girdler, Angelica Cabatu, Hailey Olds, Geoffrey A Potts

**Affiliations:** 1 Dermatology, Wayne State University School of Medicine, Detroit, USA; 2 Dermatology, Wayne State University, Detroit, USA

**Keywords:** acne, androgenic alopecia, castor oil, contact dermatitis, dermatology, drug penetration, hair quality, hyperpigmentation

## Abstract

Castor oil, derived from the seeds of *Ricinus communis*, has a long history of use in traditional medicine and cosmetics. This review examines the current dermatological applications of castor oil, highlighting both its therapeutic potential and safety profile. A comprehensive literature search was conducted using PubMed and Google Scholar to identify studies evaluating castor oil in the treatment or enhancement of treatments for various dermatologic conditions. Evidence supports its use in hair care for improving luster and possibly combating androgenic alopecia via inhibition of prostaglandin D2 synthesis. This review also highlights the efficacy of castor oil in reducing hyperpigmentation and improving hydration, elasticity, and signs of aging through its antioxidant properties. Additionally, castor oil has been added to various formulations to increase drug penetration and cleansing efficacy with fewer irritant effects. Though generally well tolerated, rare adverse effects such as contact dermatitis and hair felting have been reported. Castor oil shows promise as a versatile and accessible agent in dermatology, warranting further clinical investigation to fully establish its efficacy and optimal use.

## Introduction and background

Introduction 

Castor oil is a vegetable oil derived from the seeds of *Ricinus communis*, a plant native to northeastern Africa and India [1]. The plant is now grown globally in tropical and subtropical regions such as India, Brazil, and China, with India being the leading producer and exporter of castor oil [1]. The oil itself is composed primarily of ricinoleic acid, a monounsaturated omega-9 fatty acid that constitutes approximately 90% of its fatty acid content [1]. This high ricinoleic acid content is largely responsible for castor oil’s distinctive chemical profile and its biological activities, including anti-inflammatory, antimicrobial, and antioxidant properties [1]. It is important to distinguish native castor oil from its chemically modified derivatives, which are frequently used in dermatologic and cosmetic formulations. Native castor oil consists primarily of triglycerides rich in ricinoleic acid, whereas hydrogenated castor oil, polyethylene glycol (PEG)-castor oil derivatives, and castor oil-based polyesters are structurally altered to improve stability, emulsification, or penetration, and often function as excipients rather than primary therapeutic agents. Castor oil has gained renewed relevance amid increasing demand for clean-label, barrier-friendly formulations and dermatologic therapies that reflect the needs and practices of diverse skin and hair types. 

Historically, castor oil has held significant cultural and medicinal importance across various societies [2]. In African, Caribbean, and Indigenous American traditions, castor oil has been a staple in hair and scalp care, particularly among communities with textured hair types [2]. Its conditioning properties have been used to enhance hair luster and promote manageability, and many cultures still continue to use castor oil for these purposes today [3].

In dermatology, castor oil has traditionally been employed as a moisturizing agent and as a carrier oil in topical formulations. However, recent research has begun to systematically explore its potential therapeutic roles in a range of skin and hair disorders. These include acne, melasma, hyperpigmentation, androgenic alopecia, psoriasis, wound healing, and skin aging [4-17]. Additionally, castor oil has shown utility in improving the dermal penetration of active pharmaceutical ingredients and may replace more abrasive or irritating components in topical cleansers[4,14]. Purified castor oil does not contain ricin, a known toxin found in the seeds of castor oil plants [1]; however, rare allergic reactions have been reported and are discussed herein.

Despite its broad use in both traditional and modern settings, castor oil remains relatively unexplored in the dermatologic literature. There is no contemporary dermatology-focused narrative review that synthesizes clinical, mechanistic, formulation-based, and safety data while distinguishing castor oil from its derivatives. Much of its usage is supported by anecdotal evidence or extrapolated from preclinical findings. This narrative review aims to synthesize the current scientific literature evaluating castor oil’s role in dermatology. 

Search methods

This manuscript is a narrative review conducted in accordance with the Scale for the Assessment of Narrative Review Articles (SANRA) to ensure clarity, transparency, and scientific rigor. Given the heterogeneity of study designs and outcomes, quantitative synthesis was not performed; instead, findings were synthesized using principles outlined in the Synthesis Without Meta-Analysis (SWiM) reporting guideline. A comprehensive literature search was conducted using PubMed, Google Scholar, MEDLINE, and Cochrane Library to identify peer-reviewed articles evaluating the dermatologic use of castor oil and castor oil-derived compounds. Searches included articles published up to April 20, 2025. The following search terms and Boolean operators were used in various combinations: “castor oil” OR “ricinoleic acid” OR “hydrogenated castor oil” AND “dermatology” OR “skin” OR “hair” OR “alopecia” OR “acne” OR “melasma” OR “hyperpigmentation” OR “wound healing” OR “psoriasis” OR “onychomycosis”. 

Two independent researchers gathered and evaluated resources relevant to the topic. Reference lists of relevant articles were also manually screened to identify additional pertinent studies. Studies were eligible for inclusion if they evaluated castor oil or castor oil-derived compounds and addressed dermatologic or hair-related indications, including clinical, cosmetic, formulation-based, or mechanistic outcomes. Excluded studies included articles unrelated to dermatology or skin, hair, or scalp conditions, or if they were from non-scientific sources without primary data or clear relevance. Studies evaluating formulations in which the role of castor oil could not be reasonably ascertained were also excluded. Resources were not included if they were not published in English. Both clinical and preclinical studies were included due to the limited availability of large randomized controlled trials in this area. Abstracts were screened for relevance, followed by full-text review when available. In instances where full texts were inaccessible, abstracts were included if they provided sufficient methodological and outcome detail relevant to the scope of the review.

Given the narrative nature of this review and the diversity of included study designs (ranging from in vitro studies and formulation research to clinical trials and case reports), formal risk-of-bias tools were not uniformly applied. Instead, study limitations, sample size, design strength, and applicability were qualitatively assessed. 

Where applicable, outcomes were contextualized according to the hierarchy of evidence, with distinctions made between preclinical findings, early clinical studies, randomized trials, and case reports. Results were synthesized narratively in accordance with SWiM principles, grouping studies by dermatologic indication and thematic relevance. No statistical pooling or meta-analysis was performed due to heterogeneity in study populations, interventions, comparators, and outcome measures.

## Review

Dermatologic uses of castor oil

Hair Luster and Quality 

The use of hair oils, including castor oil, is a deep-rooted cultural practice used by many in African and Indian cultures as well as in other communities with skin of color [3]. Most often, the use of castor oil is thought to maintain hair moisture and luster, treat scalp dryness or pruritus, and make the hair more manageable to style [2]. While many of these claims are cultural practices and have not been studied to show clinical efficacy, there have been a few studies investigating the use of castor oil for hair health. One study evaluated the effect of a castor oil treatment on the luster or light reflection on varying hair types using goniophotometer scattering curves. Castor oil was found to increase the contrast between strands of hair, suggesting an increase in the luster or shine of the hair strands [15]. 

Androgenic Alopecia 

Recent studies by Garza et al. have shown that prostaglandin D_2_ (PGD_2_) naturally inhibits hair growth and is found in higher concentrations on the scalp of those suffering from androgenic alopecia (AGA) [7]. In a preclinical experimental investigation, researchers found that ricinoleic acid (the active ingredient of castor oil) binds and inhibits the prostaglandin D_2_ synthase (PTGDS) enzyme responsible for producing PGD_2_, and thus may be a therapeutic option for hair loss [7]. Although topical application of oils may lead to limited systemic absorption of small volatile constituents, evidence indicates that the majority of lipid components remain localized within the stratum corneum, with minimal penetration into systemic circulation [16]. Given that PGD_2_ levels are elevated locally within the scalp in AGA, the lack of systemic absorption does not represent a limitation for topical treatment. In a study by Fong et al., ricinoleic acid did not cause any adverse skin reactions or skin toxicity [16]. This highlights the potential for castor oil to be used in AGA and other forms of hair loss with elevated PGD_2_ levels, and its favorable safety profile. Although ricinoleic acid has been shown to inhibit prostaglandin D_2_ synthase, the clinical translation of PGD_2_ pathway modulation in androgenetic alopecia remains uncertain. A randomized, double-blind, placebo-controlled trial of setipiprant, a PGD_2_ receptor antagonist, demonstrated no clinical efficacy in men with androgenetic alopecia. This finding highlights limitations in direct translation of this pathway and the need for further investigation before castor oil-based interventions for androgenetic alopecia can be established [18]. 

Acne Vulgaris

Acne vulgaris is a common, chronic skin condition involving increased bacterial colonization, sebum production, and inflammation of hair follicles [19]. Adapalene gel is a common topical treatment for this condition, but it has low percutaneous penetration, which may limit treatment efficacy [4]. One study aimed to improve the penetration, retention, and bioavailability of adapalene via microemulsion. A component of this formulation was hydrogenated castor oil, along with isopropyl myristate, ethanol, and water in a fixed ratio to adapalene [4]. The abstract did not report the concentration of hydrogenated castor oil or the full composition of the comparator vehicle, limiting detailed formulation-level interpretation. They found that their formulation increased dermal retention, epidermal penetration, local bioavailability, and efficacy compared to Differin^®^, a brand-name adapalene formulation (Galderma Laboratories, Zug, Switzerland) [4]. 

Hyperpigmentation

Infraorbital hyperpigmentation, otherwise known as ‘dark under eyes’, is a common reason for many patients seeking dermatologic care [6]. In a single-arm clinical trial, castor oil cream was found to significantly decrease the amount of melanin, erythema, and skin laxity in the infraorbital area after 2 months of use [6]. Changes in skin color and pigmentation were measured using the VisioFace® skin analysis device (Courage + Khazaka Electronic GmbH, Cologne, Germany) and showed a statistically significant decrease of 5 points after the 2-month trial of castor oil cream [6].

Melasma is another common skin condition most often occurring in women and patients of darker skin types [17]. It is characterized by symmetrical hyperpigmentation of sun-exposed areas, most commonly on the face, and may be triggered by pregnancy [17]. A prospective, non-randomized, uncontrolled interventional clinical study by Piamphongsant evaluated the safety and efficacy of a phenol-castor oil peel in patients with deep melasma [5]. Castor oil was used as a non-toxic carrier in the phenol solution, and the optimal formula (Formula 4, with 0.5% castor oil) induced moderate inflammation and effective exfoliation with fewer side effects than traditional croton oil-based peels [5].

It is important to note that castor oil acts as the carrying substrate for the phenol, which is the active ingredient responsible for the depigmentation effect. In particular, the phenol-castor oil showed no evidence of cardiac arrhythmias in all patients, which is a possible adverse effect of croton-phenol peels [5]. Thus, no cardiac monitoring after the procedure is needed, as it is with croton-phenol peels [5]. In the study, this formula led to a significant pigment reduction, and the average melasma indices, measured by a DermaSpectrometer^®^ (Courage + Khazaka Electronic GmbH, Cologne, Germany), dropped from 206.4 to 91.2 across 30 patients [5]. Some even experienced complete clearance within one week [5]. The study concluded that the short 1-minute application of the phenol-castor oil peel is a safe and effective option for melasma treatment when combined with topical therapy afterwards [5].

Anti-Aging and Combating Reactive Oxygen Species

Free radicals are unstable molecules that can damage skin cells and accelerate aging, leading to wrinkles, fine lines, and loss of elasticity [20]. Antioxidants can neutralize these free radicals and thus treat and prevent these signs of aging [20]. Researchers compared the antioxidant effects of castor oil to ascorbic acid (commonly known as vitamin C), which is an ingredient commonly used for treating UV damage and hyperpigmentation [20]. The median inhibitory concentration (IC50) value of both substances was compared in a 2,2-diphenyl-1-picrylhydrazyl (DPPH) radical scavenging test [13]. The IC_50_ of castor oil was 19.02 µg/mL, compared to vitamin C, which was 2.36 µg/mL [13]. This qualifies castor oil as a strong antioxidant with properties that allow it to protect the skin from reactive oxygen species and premature aging [13].

Small wrinkles around the eye area, also known as periorbital rhytides, are a common first sign of aging [21]. Researchers incidentally discovered that castor oil significantly decreased the appearance of rhytides around the eye [6]. Application two times daily for two months led to a 33% improvement (2.45 to 1.64), and the appearance of fine lines and wrinkles was visibly decreased [6]. This further highlights the potential utility of castor oil for signs of aging.

Skin Elasticity

Over time, as people age, their skin becomes less elastic and resistant to stretch and stress [21]. This can lead to signs of wrinkles, sagging skin, and other signs of damage [21]. One short-term experimental application study analyzed the effect of two lotions on skin elasticity and softness [20]. The test lotion contained quaternary ammonium salts of triglycerides made from castor oil, and the control lotion was the same lotion without these castor oil salts [22]. The results showed an increase in skin elasticity two hours after the application, whereas the control lotion only showed an increase in elasticity immediately after application [22]. While a noticeable difference in elasticity two hours post-treatment is significant, there were no results for long-term efficacy, creating a need for further investigation. This study also showed that the areas treated with the castor oil lotion demonstrated a reduction in mechanical resistance and dynamic viscosity, which correlates with softening of the skin and increased skin elasticity [22]. The results of this study highlight the possible benefits of castor oil in cosmetic lotions and moisturizers to further improve the look of skin and increase elasticity.

Skin Cleansing

In occupations and situations where one’s skin is soiled with grease, oil, coal, and other oily substances, a strong cleaning product is typically needed to clean the skin [14]. Most effective cleansers include abrasive substances such as walnut shell, corn, pumice, or plastic to help cut through the grease and oil; however, this can leave the skin irritated and dry [14]. A randomized controlled double-blind study found that adding hydrogenated castor oil beads to detergent, compared to walnut shell, achieved the same efficacy of cleanliness but with significantly less redness and irritation [14]. The study tested both substances in addition to detergent alone in those with normal skin texture and those with atopic dermatitis [14]. The castor oil beads were found to be helpful in atopic skin as they preserved the skin barrier significantly better (as approximated through transepidermal water loss) [14]. This shows that castor oil can be a powerful adjunct to a cleanser to preserve hydration with less irritation, especially in the setting of atopic skin.

Wound Healing

Castor oil is also frequently used in wound dressing due to its capability to promote oxygen permeability, which aids epithelial cell growth and collagen filament production, and its ability to decrease the loss of water vapor from the wound [12]. Furthermore, polyesters made from castor oil have been found to demonstrate antimicrobial activity against *Escherichia coli *and *Staphylococcus aureus*, which further exemplifies their potential in wound dressing materials [11].

Castor oil may also be beneficial in healing skin graft donor sites. In a 6-month study, castor oil-balsam of Peru-trypsin ointment (Xenaderm™, HealthPoint, Fort Worth, USA) was applied and dressed with non-adherent gauze on chronic wounds with split-thickness skin grafts harvested from the thighs and applied to the lower legs or foot from the same leg [10]. After 11 days, all donor site wounds were epithelialized, and common complications such as excessive pain, bleeding, infection, and foul odor were not observed [10].

Similarly, Xenaderm was used to treat pressure ulcers of long-term care residents in another study. Their findings suggest that the use of Xenaderm leads to shorter healing times and higher healing rates in stage 1 and 2 ulcers, although the results were not significant, possibly due to the variance of treatment times [23]. The success of Xenaderm is further exemplified by a case report of bullous lesions that were cleansed gently with saline, followed by an application of Xenaderm twice daily and covered by oil emulsion-impregnated gauze [24]. The open blisters decreased in size and stopped draining, while closed blisters began to shrink within a week [24]. Full healing resulted after one week, and the patient endorsed no pain or discomfort during the treatment [24].

Similarly, another case report used an analogous protocol in a woman who developed a wet desquamation reaction after chemotherapy and radiation therapy for breast cancer [24]. After 7 days, she endorsed less irritation, pain, and drainage without any burning sensation [25]. By two weeks, her wound completely healed, and she discontinued treatment [25].

Chronic Pruritus

The skin is a vital organ of the human body, with one of its main functions being a protective barrier to the external environment [26]. Itching is a common complaint and may be due to irritating substances coming into contact with the skin. However, the pathogenesis of the itch can differ and involve numerous receptors and mediators. Capsaicinoids, which often inflict pain and itchiness upon initial application, eventually bring relief and are an option for treating chronic pruritus [26]. One study aimed to formulate a treatment that has increased substantivity to reduce the number of applications, increase compliance, and create a more effective treatment for chronic pruritus. They created an oil-in-oil emulsion with polydimethylsiloxane due to its water repellent properties and castor oil, which is lipophilic, to incorporate nonivamide, a synthetic analogue of capsaicin, in the dispersed phase [26]. These oil-in-oil emulsions appear to be superior to semi-solid formulations, as permeation was constant over a period of 10 hours, bringing longer-lasting relief [26].

Onychomycosis

Onychomycosis is a fungal infection of the nails [27]. It can be difficult to treat, because topical medications must penetrate the nail plate for successful treatment [27]. Researchers evaluated the effectiveness of Miconal Nails® (Morgan Pharma, Monteviale, Italy), a new topical lacquer containing hydrogenated castor oil, hydroxyethyl cellulose, urea, climbazole, piroctone olamine, and undecylenic acid [27]. In a single-arm clinical trial of 25 patients with ages ranging from 20 to 70 years old, 15 patients (60%) achieved complete resolution, and seven (28%) had improvement [27]. While not yet available for public consumption in the United States, this new product may serve as an effective approach to treating onychomycosis in the future, using castor oil as a vehicle to enhance penetration of active ingredients.

Topical Formulations

Topical medications are used for a variety of dermatologic conditions, including acne treatment, infection, and skin pigmentation. A problem often encountered is the lack of penetration, either due to solubility or stability. Creating solutions and emulsions with castor oil can help solve these issues, improving drug delivery. For the treatment of hyperpigmentation, resveratrol inhibits tyrosinase to decrease melanin production [28]. It is created in a microemulsion gel format using castor oil as the emulsifier [28]. This combination increased the transdermal penetration and rate significantly while still retaining resveratrol’s ability to combat hyperpigmentation [28].

Similarly, psoriasis is an inflammatory disease of the skin that may also involve the nails and joints [9]. Topically, the first-line treatment is a topical steroid ointment, which has a petroleum jelly base [9]. However, some patients do not like the texture, and it may stain clothing, both of which can lead to poor patient compliance. Moreover, the active ingredients sometimes do not penetrate the thickened epidermis enough to be effective [9]. Researchers aimed to create a formulation that was semi-solid and demonstrated higher substantivity on skin to increase penetrance of betamethasone dipropionate and calcipotriol [9]. They elected to create an oleogel composed of castor oil, medium-chain fatty acids, and film-former OleoCraft™ MP-30 (Croda Beauty, Snaith, East Yorkshire, United Kingdom) [9]. Their formulation demonstrated significant substantivity compared to marketed products but resulted in decreased penetration of betamethasone dipropionate [9]. This study, however, introduces the value of using lipophilic drugs for topical treatment in comparison to petroleum jelly to increase patient compliance and decrease the number of applications [9].

Adverse effects

Contact Dermatitis 

The use of castor oil may come with a few dermatologic adverse reactions. Most commonly, patients encounter contact dermatitis to castor oil in cosmetic and hygiene products such as makeup, shampoo, and deodorant [29,30]. This is rare but may still occur. One study reported a case of axillary dermatitis after using a deodorant containing hydrogenated castor oil [29]. On patch testing, the patient reacted to undiluted castor oil but not when diluted with petrolatum [29]. When 10 control patients were patch tested with the same undiluted castor oil, they showed no reaction, highlighting the fact that the reaction was specific to the individual, rather than a general irritant [30].

Another case report detailed a woman who developed itching, darkening, and swelling of her lips for over a year [30]. On a physical exam, she had erythema, scaling, and hyperpigmentation of the upper and lower lips [30]. The patient was prescribed topical corticosteroids and underwent patch testing and had a positive skin reaction on the patch test to the 30% ricinoleic acid (the main ingredient of castor oil) as well as to many commercial lipsticks [30]. The patient stopped using all lipstick products for a year, and her symptoms resolved, except for residual hyperpigmentation [30]. This case study showed another example of contact dermatitis likely due to castor oil within common cosmetic products [30].

In yet another case report, a patient presented with diffuse facial swelling and urticaria-like lesions on their back [31]. This developed within hours of the patient using Otocerum® (Laboratorio Reig Jofre, Barcelona, Spain) in their ear for ear wax removal [31]. Skin patch testing of Otocerum® in this patient was positive [31]. Further patch testing was performed with all the ingredients of Otocerum® separately, and the patient only reacted to castor oil [31]. This further demonstrates that castor oil may cause contact dermatitis, but also angioedema-like symptoms in some patients [31].

As mentioned previously, castor oil is widely used to help with wound healing in various forms of dressings [22]. In one case report, a man developed allergic contact dermatitis to a dressing containing castor oil [32]. The patient then underwent subsequent patch testing to many forms of castor oil, including both hydrogenated and non-hydrogenated castor oil [32]. He reacted to only non-hydrogenated forms of castor oil [32]. Further analysis showed that the non-hydrogenated castor oil contained ricinoleic acid, whereas hydrogenated castor oil did not [32]. This study suggests that ricinoleic acid is most likely the inciting agent for contact dermatitis [32].

Across reported cases, adverse reactions have most commonly been associated with non-hydrogenated castor oil, with evidence suggesting that ricinoleic acid is the primary sensitizing component, whereas hydrogenated and PEG-modified castor oil derivatives demonstrate a lower risk of allergic contact dermatitis. While contact dermatitis is the most common adverse effect of castor oil use, it is fortunately typically mild and can be treated quickly. Stopping use of the offending agent typically relieves the rash, but topical corticosteroids or antihistamines can be used as well [33].

Hair Felting

Hair felting is a rare condition in which the hair suddenly mats and becomes tangled, twisted, and impossible to comb through [34]. One case reported that a patient developed this immediately after applying castor oil to the hair for the first time [34]. It is a devastating event and requires the hair to be cut off as it cannot be de-tangled or reversed [34]. Hair felting can also cause severe psychological distress as the acute nature and loss of hair affects many people very deeply [34]. It is important to keep in mind that, very rarely, castor oil can trigger this.

Safety Summary

Overall, castor oil demonstrates a favorable safety profile when used topically, particularly in hydrogenated or chemically modified forms. Cosmetic Ingredient Review (CIR) expert panel assessments have concluded that PEG-castor oil derivatives are safe for use in cosmetic formulations at current concentrations, with a low incidence of skin sensitization and irritation reported in human and animal studies [35]. Adverse reactions are rare, typically mild, and most strongly associated with native castor oil and ricinoleic acid, underscoring the importance of formulation-specific considerations in dermatologic practice.

## Conclusions

Castor oil is an easily accessible vegetable oil, with many studies showing its usefulness for various dermatologic conditions. These include hair loss and quality, hyperpigmentation, acne, wound healing, anti-aging, and psoriasis. Castor oil has also been used for skin cleansing, onychomycosis, and increasing the penetration of active ingredients in topical formulations. Fortunately, adverse effects are rare but may include contact dermatitis and hair felting. Castor oil has anti-inflammatory, antimicrobial, and antioxidative properties, and has a reliable safety profile. Even though current data are favorable, further research should be done to define the long-term safety, efficacy, and optimal formulations for castor oil in dermatology. Castor oil should be considered a safe and accessible ingredient for use in dermatology today.
